# Physical performance in relation to preserved ratio impaired spirometry: a cross-sectional study of community-dwelling older Japanese adults

**DOI:** 10.1038/s41598-021-96830-6

**Published:** 2021-08-31

**Authors:** Kunihiko Anami, Shin Murata, Hideki Nakano, Koji Nonaka, Hiroaki Iwase, Kayoko Shiraiwa, Teppei Abiko, Akio Goda, Jun Horie

**Affiliations:** 1 Department of Rehabilitation, Faculty of Health Sciences, Naragakuen University, 3-15-1, Nakatomigaoka, Nara-shi, Nara, 631-8524 Japan; 2 Department of Physical Therapy, Faculty of Health Sciences, Kyoto Tachibana University, Kyoto, 607-8175 Japan; 3 Department of Physical Therapy, Faculty of Rehabilitation, Kobe International University, Kobe, 658-0032 Japan

**Keywords:** Health care, Medical research

## Abstract

Preserved ratio impaired spirometry (PRISm) is associated with an increased mortality rate; however, its characteristics have not been clearly identified in Japan. This cross-sectional study of community-dwelling older adults compared physical function between people with PRISm and those with no respiratory issues, from 2014 to 2019. We collected demographic data through interviews and measured respiratory and physical functions. We included 668 older adults (male, 23.5%; mean age, 72.8 ± 5.6 years); the prevalence of PRISm was 12%, while the prevalence of obstruction was 6.9%. Propensity score matching was used to identify control subjects with normal spirometry (n = 80) while minimizing the effects of confounders during comparisons with the PRISm population (n = 80). Compared with community-dwelling older adults with normal lung capacity, older adults with PRISm had a lower forced vital capacity (%FVC; 68.7 ± 9.1% vs. 92.5 ± 12.7%, *p* < 0.001), lower core muscle endurance (sit-up test: 6.7 ± 5.8 vs. 8.7 ± 6.0, *p* = 0.032), and a longer one-leg stance duration (52.4 ± 41.1 s vs. 36.4 ± 34.1 s, *p* = 0.008). In multivariable logistic regression, %FVC and increased one-leg stance were independent predictors of PRISm status. The prevalence of PRISm among community-dwelling elderly Japanese exceeds that of obstructive lung disease and is associated with reduced %FVC and better performance on balance testing.

## Introduction

Preserved ratio impaired spirometry (PRISm), generally known as the ‘restrictive ventilatory defect’, refers to a condition in which spirometry shows a ratio of forced expiratory volume in the first second (FEV_1_) to forced vital capacity (FVC) of ≥ 70% and a predicted FEV_1_ (%FEV_1_) of < 80%^1^. The prevalence of PRISm has been reported to be between 12 and 24%^1–4^. Its association with respiratory symptoms^3,5–9^ and increased mortality^2,9–12^ means that PRISm cannot be ignored in clinical settings^13,14^. Risk factors include age, female sex, a history of smoking, diabetes mellitus, and obesity^5,7,15,16^. Moreover, increased dyspnoea, reduced 6-min walk distance^1^, and reduced health-related quality of life^17,18^ have been reported in individuals with PRISm.

The Genetic Epidemiology of COPD (COPDGene) study and other reports discussing PRISm^1–3,8,18^ have explained the condition using medical data^4,6,9^. However, few studies have focused on community-dwelling individuals^7^. In Japan, awareness of PRISm remains low, and its prevalence is unknown. To date, no community-based studies have been conducted.

Past research has reported the prevalence of chronic obstructive pulmonary disease (COPD) in community-dwelling persons^19,20^, as well as physical function and cognitive function characteristics of community-dwelling individuals suspected of having COPD^21–25^. Physical characteristics of PRISm, such as reduced lung capacity and being overweight^5,7,16^, have also been elucidated but, aside from a reduced 6-min walk distance^1^, physical function characteristics remain unexplored. Previous studies have found that individuals with PRISm have a shorter 6-min walk distance; therefore, we hypothesised that PRISm would also be associated with a decline in physical function, such as reduced limb skeletal muscle strength and flexibility. If patients with PRISm had poorer physical function than individuals with normal respiratory function, it would be possible to use physical function parameters and physical characteristics as early warning signs of PRISm.

Therefore, in this study, we aimed to clarify the prevalence of hidden PRISm in Japan and to investigate differences in terms of attributes and physical function between community-dwelling older adults with normal respiratory function and those with PRISm.

## Results

**Figure 1:**
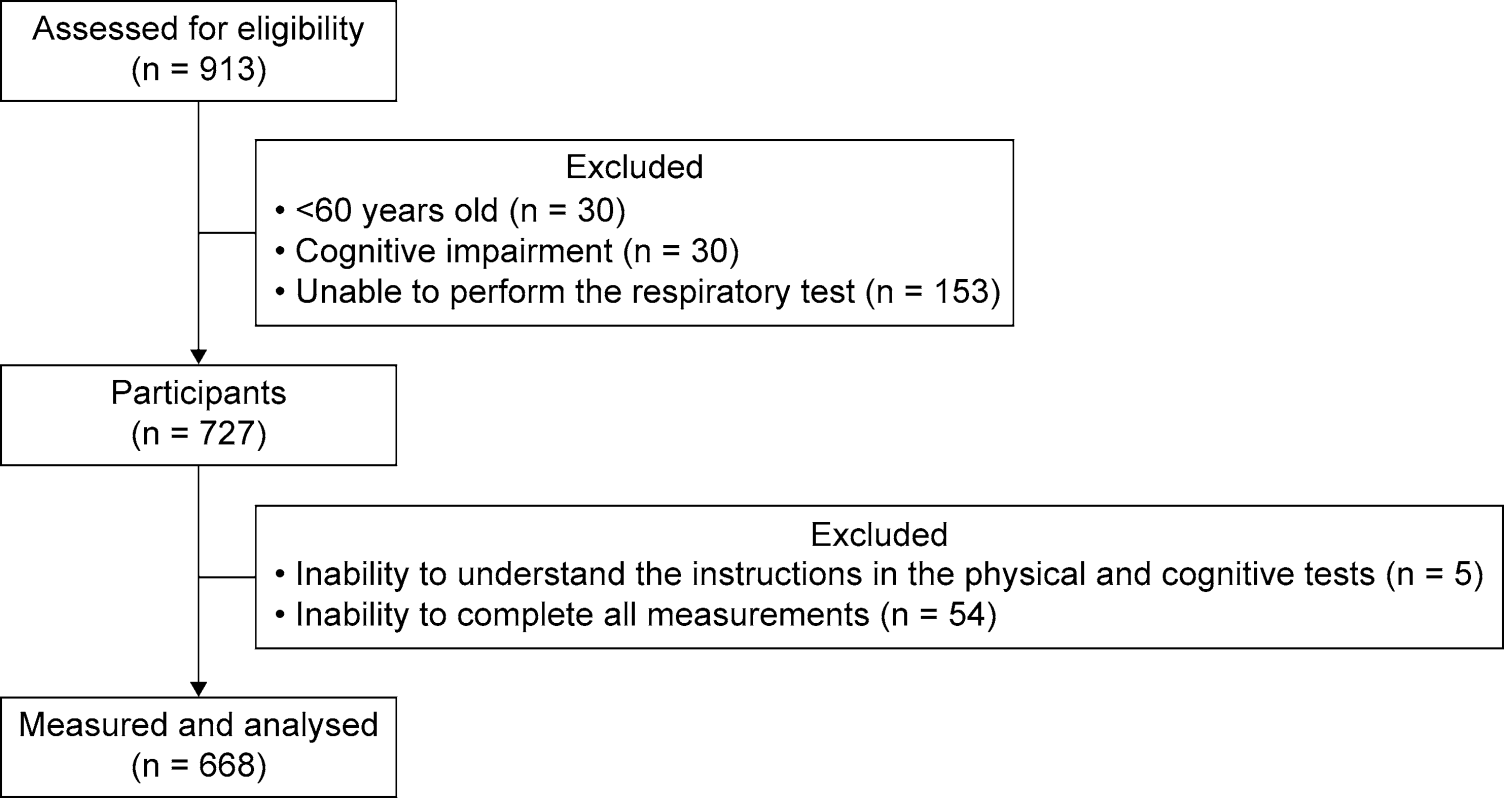
Flowchart illustrating selection of study participants.

A total of 668 community-dwelling older adults were included in this study (Fig. 1). The mean age of the participants was 72.8 ± 5.6 years. There were 157 males (23.5%) and 511 females (76.5%). The PRISm group comprised 80 participants (12.0%), the normal respiratory function group comprised 542 participants (81.1%), and there were 46 participants (6.9%) with obstructive ventilation. The study participants’ attributes are shown in Tables 1 and 2. Propensity score matching was used to eliminate confounding bias in relation to community-dwelling older adults with PRISm and those with normal respiratory function, and the two groups were compared. The results of the comparison of attributes between the two groups are shown in Table 3, and physical function measurements are shown in Table 4. Significant between-group differences were found for FVC (*p* < 0.001), %FVC (*p* < 0.001), FEV (*p* < 0.001), %FEV (*p* < 0.001), FEV/FVC (*p* = 0.041), the sit-up test (*p* = 0.032), and the one-leg stance test (*p* = 0.008). No significant differences were found in terms of diabetes mellitus, respiratory disease, and any of the other items.

**Table 1:** Sociodemographic and clinical characteristics of the PRISm, spirometric obstruction, and normal spirometry groups.

	Total	PRISm	Normal spirometry	Spirometric obstruction
	(n = 668)	(n = 80, 12.0%)	(n = 542, 81.1%)	(n = 46, 6.9%)
Age, years	72.8 ± 5.6	72.0 ± 5.1	72.8 ± 5.6	73.5 ± 5.8
**Sex**				
Male, n (%)	157 (23.5%)	15 (18.8%)	130 (24.0%)	12 (26.1%)
Female, n (%)	511 (76.5%)	65 (81.3%)	412 (76.0%)	34 (73.9%)
Education, years	12 ± 2.2	11.9 ± 2.3	12.0 ± 2.2	11.8 ± 2.3
Current and former smokers, yes, n (%)	116 (17.4%)	23 (28.8%)	75 (13.8%)	18 (39.1%)
**Diseases**				
Hypertension, yes, n (%)	235 (35.2%)	34 (42.5%)	188 (34.7%)	13 (28.3%)
Dyslipidemia, yes, n (%)	81 (12.1%)	8 (10.0%)	67 (12.4%)	6 (13.0%)
Diabetes, yes, n (%)	113 (16.9%)	12 (15.0%)	93 (17.2%)	8 (17.4%)
Cardiovascular disease, yes, n (%)	45 (6.7%)	6 (7.5%)	31 (5.7%)	8 (17.4%)
Cancer, yes, n (%)	13 (1.9%)	3 (3.8%)	8 (1.5%)	2 (4.3%)
Osteoporosis, yes, n (%)	34 (5.1%)	6 (7.5%)	28 (5.2%)	0 (0.0%)
Orthopaedic disease, yes, n (%)	58 (8.7%)	10 (12.5%)	45 (8.3%)	3 (6.5%)
Pulmonary disease, yes, n (%)	13 (1.9%)	0 (0.0%)	8 (1.5%)	5 (10.9%)
Other, yes, n (%)	81 (12.1%)	11 (13.8%)	63 (11.6%)	7 (15.2%)

*PRISm* Preserved ratio impaired spirometry.

**Table 2:** Physiological characteristics of the PRISm, spirometric obstruction, and normal spirometry groups.

	Total (n = 668)	PRISm (n = 80, 12.0%)	Normal spirometry (n = 542, 81.1%)	Spirometric obstruction (n = 46, 6.9%)
MMSE, score	28.0 ± 1.9	28.2 ± 1.7	28.1 ± 1.9	27.2 ± 2.0
Height, cm	154.3 ± 7.8	153.8 ± 7.3	154.4 ± 7.8	153.7 ± 8.4
Weight, kg	53.4 ± 8.9	53.1 ± 8.4	53.4 ± 9.0	54.0 ± 8.3
BMI, kg/cm^2^	22.4 ± 3.1	22.4 ± 3.0	22.3 ± 3.1	22.8 ± 2.9
Fat mass, kg	15.4 ± 5.3	16.1 ± 5.1	15.4 ± 5.2	13.3 ± 5.3
FMI, kg/cm^2^	6.5 ± 2.3	6.8 ± 2.2	6.5 ± 2.3	5.7 ± 2.5
Skeletal mass, kg	20.2 ± 4.0	21.2 ± 4.6	20.0 ± 3.8	20.9 ± 4.7
SMI, kg/cm^2^	8.4 ± 1.0	8.6 ± 1.2	8.4 ± 1.0	8.4 ± 1.0
FVC, L	2.32 ± 0.61	1.93 ± 0.56	2.37 ± 0.59	2.42 ± 0.76
%FVC, %predicted	89.1 ± 14.8	68.7 ± 9.1	92.3 ± 12.8	87.2 ± 16.5
FEV, L	1.82 ± 0.48	1.50 ± 0.42	1.89 ± 0.46	1.53 ± 0.56
%FEV, %predicted	94.9 ± 17.4	71.3 ± 7.3	100.0 ± 13.9	74.7 ± 19.3
FEV/FVC, %	78.8 ± 7.1	78.3 ± 6.1	80.2 ± 5.3	62.6 ± 7.8
Grip strength, kg	26.5 ± 7.0	26.0 ± 6.5	26.7 ± 7.0	25.8 ± 7.1
Knee extension strength, %	39.1 ± 10.7	40.0 ± 10.6	38.9 ± 10.8	39.9 ± 9.8
Toe-grip strength, kg	9.4 ± 4.6	9.7 ± 4.8	9.3 ± 4.4	10.5 ± 5.7
Sit-up test, score	7.7 ± 6.5	6.7 ± 5.8	7.9 ± 6.6	6.8 ± 5.7
CS-30, score	20.3 ± 5.7	20.3 ± 5.5	20.3 ± 5.7	20.3 ± 5.4
Sit-and-reach test, cm	34.4 ± 9.2	35.1 ± 9.7	34.3 ± 9.3	34.4 ± 7.6
One-leg stance test, s	47.9 ± 40.2	52.4 ± 41.1	47.6 ± 40.3	44.2 ± 37.6
Fastest gait speed, m/s	1.96 ± 0.36	1.96 ± 0.35	1.96 ± 0.36	1.93 ± 0.41
TUG, s	5.89 ± 1.20	5.87 ± 1.20	5.89 ± 1.22	5.94 ± 1.16

*BMI* Body mass index, *CS-30* 30-s stand test, *FEV* Forced expiratory volume, *FMI* Fat mass index, *FVC* Forced vital capacity, *MMSE* Mini-mental state examination, *PRISm* Preserved ratio impaired spirometry, *SMI* Skeletal muscle mass index, *TUG* Timed up and go.

**Table 3:** Comparison of the sociodemographic, clinical, and physiological characteristics of the PRISm and normal spirometry groups.

	PRISm	Normal spirometry	*p*	Effect size
	(n = 80)	(n = 80)		(φ, d)
Age, years	72 ± 5.1	72.8 ± 5.6	0.621	0.080
**Sex**				
Male, n (%)	15 (18.8%)	18 (22.5%)	0.558	0.046
Female, n (%)	65 (81.3%)	62 (77.5%)		
Education, years	11.9 ± 2.3	12.0 ± 2.5	0.795	0.040
Current and former smokers, yes, n (%)	23 (28.8%)	22 (27.5%)	0.860	0.014
**Diseases**				
Hypertension, yes, n (%)	34 (42.5%)	26 (32.5%)	0.191	0.103
Dyslipidemia, yes, n (%)	8 (10.0%)	10 (12.5%)	0.617	0.040
Diabetes, yes, n (%)	12 (15.0%)	7 (8.7%)	0.222	0.097
Cardiovascular disease, yes, n (%)	6 (7.5%)	6 (7.5%)	1.000	0.000
Cancer, yes, n (%)	3 (3.8%)	3 (3.8%)	1.000	0.000
Osteoporosis, yes, n (%)	6 (7.5%)	6 (7.5%)	1.000	0.000
Orthopaedic disease, yes, n (%)	10 (12.5%)	13 (16.3%)	0.499	0.053
Pulmonary disease, yes, n (%)	0 (0.0%)	2 (2.5%)	0.497	0.113
Other, yes, n (%)	11 (13.8%)	18 (22.5%)	0.151	0.114
MMSE, score	28.2 ± 1.7	28.2 ± 1.7	0.926	0.010
Height, cm	153.8 ± 7.3	154.8 ± 7.9	0.403	0.130
Weight, kg	53.1 ± 8.4	53.8 ± 8.4	0.609	0.080
BMI, kg/cm^2^	22.4 ± 3	22.4 ± 3.1	0.960	0.010
Fat mass, kg	16.1 ± 5.1	15.5 ± 4.6	0.414	0.130
FMI, kg/cm^2^	6.8 ± 2.2	6.5 ± 2.0	0.338	0.150
Skeletal mass, kg	21.2 ± 4.6	20.4 ± 4.3	0.276	0.170
SMI, kg/cm^2^	8.6 ± 1.2	8.4 ± 1.0	0.329	0.150
FVC, L	1.93 ± 0.56	2.43 ± 0.66	< 0.001	0.820
%FVC, %predicted	68.7 ± 9.1	91.1 ± 11.7	< 0.001	2.150
FEV, L	1.50 ± 0.42	1.94 ± 0.53	< 0.001	0.920
%FEV, %predicted	71.3 ± 7.3	98.3 ± 13.5	< 0.001	2.480
FEV/FVC, %	78.3 ± 6.1	80.1 ± 4.9	0.041	0.330

*BMI* Body mass index, *CS-30* 30-s stand test, *FEV* Forced expiratory volume, *FMI* Fat mass index, *FVC* Forced vital capacity, *MMSE* Mini-mental state examination, *PRISm* Preserved ratio impaired spirometry, *SMI* Skeletal muscle mass index, *TUG* Timed up and go.

**Table 4:** Comparison of physical function measurements between the PRISm and normal spirometry groups.

	PRISm (n = 80)	Normal spirometry (n = 80)	*p*	Effect size (d)
Grip strength, kg	26.0 ± 6.5	25.7 ± 6.5	0.801	0.040
Knee extension strength, %	40.0 ± 10.6	42.1 ± 10.0	0.186	0.210
Toe-grip strength, kg	9.7 ± 4.8	9.0 ± 4.1	0.357	0.150
Sit-up test, score	6.7 ± 5.8	8.7 ± 6.0	*0.032*	0.340
CS-30, score	20.3 ± 5.5	20.7 ± 5.9	0.657	0.070
Sit-and-reach test, cm	35.1 ± 9.7	33.4 ± 8.7	0.264	0.180
One-leg stance test, s	52.4 ± 41.1	36.4 ± 34.1	0.008	0.420
Fastest gait speed, m/s	1.96 ± 0.35	1.95 ± 0.25	0.917	0.020
TUG, s	5.87 ± 1.13	6.06 ± 1.02	0.258	0.180

*CS-30* 30-s stand test, *PRISm* Preserved ratio impaired spirometry, *TUG* Timed up-and-go.

Subsequently, a logistic regression analysis was performed with the PRISm group as the dependent variable and with hypertension, respiratory disease, other diseases, %FVC, knee extension strength, sit-up test, and one-leg stance test as independent variables (φ, > 0.1; d, > 0.2). The results showed significant relationships for %FVC (*p* < 0.001) and the one-leg stance test (*p* = 0.004; Table 5). The receiver operating characteristic (ROC) analysis, which was performed to determine the %FVC cut-off value, showed an area under the curve (AUC) of 0.961. The %FVC cut-off value based on the Youden index was 83.6% (sensitivity, 0.988; specificity, 0.800; Table 6).

**Table 5:** Logistic regression analysis with the presence or absence of PRISm as the dependent variable.

	B	SE	Wald	*p* value	Exp (B)	95% CI for Exp (B)	
						Lower	Upper
Hypertension, yes	− 1.197	0.779	2.361	0.124	0.302	0.066	1.391
Pulmonary disease, yes	− 21.643	24,425.733	0.000	0.999	0.000	0.000	
Other, yes	− 0.501	0.868	0.333	0.564	0.606	0.111	3.319
%FVC, %predicted	− 0.423	0.089	22.402	< 0.001	0.655	0.550	0.781
Knee extension strength, %	− 0.056	0.033	2.903	0.088	0.946	0.886	1.008
Sit-up test, score	− 0.115	0.070	2.660	0.103	0.892	0.777	1.023
One-leg stance test, s	0.031	0.011	8.214	0.004	1.031	1.010	1.053
Dependent variable: PRISm

**Table 6:** ROC curve analysis of %FVC to predict PRISm among community-dwelling older people.

	AUC (95% CI)	*p* value	YI	Cut-off	Sensitivity	Specificity
%FVC, %predicted	0.961 (0.937–0.985)	< 0.0001	0.800	83.6	0.988	0.800

*AUC* Area under the curve, *CI* Confidence interval, *FVC* Forced vital capacity, *PRISm* Preserved ratio impaired spirometry, *ROC* Receiver operating characteristic, *YI* Youden’s index (Sensitivity + Specificity − 1).

## Discussion

This study aimed to clarify the prevalence of hidden PRISm in Japan and to explore differences in attributes and physical function between community-dwelling older adults with normal respiratory function and those with PRISm. The key findings, which we consider novel and important, are as follows: the prevalence of PRISm among community-dwelling older adults in Japan was 12.0%, older adults with PRISm had significantly decreased sit-up test times (used to determine physical function), and the %FVC cut-off value for predicting PRISm was 83.6%.

Previous studies have reported prevalence rates for PRISm as between 12 and 24%^1–4^. In this study, the prevalence rate was 12.0%, which was similar to that reported in some other countries. However, the community-dwelling older adults who participated in this study were participants in a health-support project sponsored by the local municipality; therefore, they were likely to have had a relatively high level of health awareness. Smoking rates and, therefore, PRISm prevalence, in Japan, may well have been higher if a larger sample (including those with low health awareness) had been surveyed, which needs to be considered in further studies in Japan. In this study, the percentage of patients with spirometric obstruction was 6.9%, with a higher percentage found in the PRISm group. We found a greater prevalence of PRISm compared with spirometric obstruction among community-dwelling older adults, which was a novel finding. Additionally, 13 participants had respiratory disease, none of whom were in the PRISm group. Based on these results, it would appear that the presence of respiratory disease is not a risk factor for PRISm.

After comparing the PRISm group with the normal respiratory function group, significant between-group differences for each indicator of respiratory function were found and, in terms of physical function, the PRISm group showed a significant decrease in sit-up test times. Wan et al.^1^ found that the 6-min walk distance drops significantly for individuals with PRISm. As such, we hypothesised that if individuals with PRISm had a shorter 6-min walk distance, a decline in physical function in relation to limb skeletal muscle strength and core muscle strength would also likely be observed. We identified no significant differences in indicators of limb skeletal muscle power, such as grip strength, or in performance, such as walking speed; however, as noted, the PRISm group showed a significant decrease in sit-up test times. The sit-up test is also considered useful for assessing core muscle endurance^26^. In this study, we were unable to undertake a 6-min walk test due to setting issues. However, this significant decrease in sit-up test times (an assessment of core muscle endurance) suggested that those with PRISm had reduced endurance. The PRISm group had significantly better one-leg stance test results. Participants in Bohannon et al.’s study^27^ undertook a one-leg stance test five times and the measurements were recorded. They found that the mean stance time was 14.2 ± 9.3 s for participants in their 70 s and 22.5 ± 8.6 s for those in their 60 s. Although the one-leg stance test results obtained in our study were higher than those reported by Bohannon et al., we do not consider the difference to be clinically important. These findings suggest that individuals with PRISm have reduced overall stamina (6-min walk distance) and core stamina but are unlikely to show reduced limb muscle strength or balance. Wan et al.^2^ found that PRISm was associated with an increased mortality rate and noted that 22.2% of the participants in their study transitioned to a diagnosis of COPD (GOLD 0), and that 25.1% had progressed to COPD (GOLD 1–4) after 5 years. Horie et al.^21^ found that community-dwelling older adults who exhibited obstructive ventilatory defects but who had not been diagnosed with COPD did not have reduced physical function compared with a group of older adults with normal respiratory function. Therefore, similar to COPD, PRISm in community-dwelling older adults is difficult to predict using physical function tests alone, and the active use of spirometry is essential^28,29^.

Previous research has identified significant differences between participants with PRISm and those with normal respiratory function regarding respiratory function and the presence or absence of a smoking history^1,5,7,15,16^. Our study findings showed that there were many older adults who smoked among those with PRISm (Table 1). Our results are consistent with those of previous studies and show that Japanese individuals follow a similar trend in this regard. Furthermore, cumulative exposure to tobacco smoke has been associated with an increased risk of death^2^. Although previous studies have found associations between PRISm and older age, female sex, body mass index (BMI), and the presence or absence of diabetes mellitus, we did not find any significant differences among these risk factors. Jones et al.’s study of the effects of BMI on lung volume^30^ reported that obesity alone did not necessarily reduce lung capacity. Our study results showed no significant difference in relation to BMI, possibly because only a small number of study participants were obese.

In this study, we investigated factors likely to be associated with PRISm, using logistic regression analysis. We found that %FVC and the one-leg stance test results were associated with PRISm. Based on these results, we calculated the %FVC cut-off value using ROC analysis. The %FVC cut-off value for predicting PRISm was 83.2%, with an AUC of 0.961, indicating very high accuracy. A sensitivity of 98.8% and a specificity of 80.0% indicated high predictive power. Although we focussed on airway obstruction, we consider that the effect of reduced FVC was also an important risk factor. These findings support the importance of spirometry in PRISm detection.

Our study findings, similar to those of previous studies, indicated that older adults with PRISm had reduced endurance. However, a lack of significant between-group differences in physical function, except for trunk endurance, is an important novel finding. Regan et al.^31^ reported that chronic smokers may exhibit clinical and physiological impairments due to smoking, even if they do not meet the diagnostic criteria for COPD. They reported that spirometry alone is likely to underestimate respiratory disease. As such, surveying physical function in addition to respiratory function and comparing it between individuals with PRISm and those with normal respiratory function is likely to be significant both clinically and in terms of social welfare generally. Moreover, conditions such as COPD and lower back pain have been identified as primary contributors to years spent living with disability, due to disease and injury sequelae^32^. Thus, it may be possible to extend healthy lifespans through early detection of PRISm, which may otherwise advance to COPD in some people, followed with appropriate medical management and health-related support.

## Limitations

This study was limited in terms of its small sample size for the logistic regression analysis, the uneven distribution of males and females, and its cross-sectional research design. Furthermore, as we recruited community-dwelling older adults, many participants were those involved in a community socialising program for older adults who did not need support. Therefore, we cannot exclude the possibility that our study participants were individuals with relatively high health awareness. Future studies are needed involving a sample of community-dwelling individuals who either do not or cannot participate in such programs. We did not use the 6-min walk test due to limited space in the study setting; therefore, a reliable endurance assessment could not be undertaken. Endurance assessments should be conducted in future studies, in the same way as in previous studies. However, it was useful to include patient-reported outcomes such as running or jogging durations.

## Conclusions

This study is the first to report the prevalence of PRISm in community-dwelling individuals in Japan (12.0%). A significant difference in core muscle endurance was found between participants with PRISm and those with normal respiratory function; however, we found no significant between-group differences in terms of other physical functions. As expected, our findings confirmed that restrictive ventilatory defect is a major issue. Our findings may facilitate health promotion planning and clinical goal setting, and serve to reinforce the importance of spirometry.

## Methods

### Participants

This study involved a cross-sectional survey of community-dwelling older adults, with annual measurements taken every September from 2014 to September 2019. Participants were recruited using flyers distributed in Yasu City, Shiga Prefecture, from June 2014 to August 2019. The flyers specified that no form of compensation would be offered for participation. Inclusion criteria comprised the following: (i) age, ≥ 60 years old; (ii) a mini-mental state examination (MMSE) score of < 24, with no apparent warning signs of cognitive impairment; and (iii) no spirometry performed after registration. Exclusion criteria comprised participants who were unable to understand the instructions for physical or cognitive tests or who were unable to complete all of the tests.

Participants ranged in age from 47 to 90 years at enrolment and included individuals with and without a history of smoking. This study was undertaken in accordance with the Declaration of Helsinki and was approved by the Ethics Committee of Kyoto Tachibana University (approval no. 19–5). Written informed consent was obtained from all participants prior to participation in the study.

After collecting data concerning age, height, and weight at a group physical assessment, the study participants completed the MMSE. Data concerning prevalence rates for hypertension, dyslipidemia, osteoporosis, orthopaedic diseases, diabetes mellitus, cardiovascular diseases, respiratory diseases, kidney diseases, and cancer were also collected from the participants. Smoking history was self-reported and included whether the participant was a current or former smoker. Finally, data from 614 study participants were analysed (Fig. 1).

### Definitions

PRISm was defined as a post-bronchodilator FEV_1_ < 80% of the predicted FEV_1_ and a ratio of FEV_1_/FVC ≥ 70%.

### Spirometry

For spirometry, the Autospiro AS-507 (Minato Medical Science, Osaka, Japan) was used to measure the flow-volume curve twice, according to the spirometry guidelines of the American Thoracic Society/European Respiratory Society^33^. Measurements included FVC, %FVC, FEV_1_, %FEV_1_, and FEV_1_/FVC.

### Body composition

To assess body composition, the InBody430 (InBody Japan Inc., Tokyo, Japan) was used to measure BMI, skeletal muscle mass, skeletal muscle mass index (SMI), body fat, and body fat percentage using the impedance method. The SMI was calculated through dividing skeletal muscle mass by height squared (kg/m^2^)^34^.

### Physical functions

#### Skeletal muscle strength

Grip strength was assessed using a digital hand dynamometer (T.K.K. 5401 Grip-D, Takei Scientific Instruments Co., Ltd., Niigata, Japan). This assessment tool has been shown to be both valid and reliable^35^. Participants were instructed to let their arms hang freely beside their bodies and stand upright so that the dynamometer did not touch them. Both sides were measured twice, and the maximum value (kg) was used.

Knee extension strength was evaluated using a handheld dynamometer (μTasF-1, Anima Corporation, Tokyo, Japan), as described in detail by Bohannon^36^. This assessment tool has been shown to be valid and reliable^37,38^. The participants were seated in a chair with the knee bent at a 90° angle, and the sensory pad was fixed to the distal end of the leg with a band. Two measurements were performed for each leg. The maximum measured value (kgf) was divided by body weight (kg) to calculate the body weight-normalised percentage (%), which was then used to represent knee extension strength.

Toe grip strength was assessed using a toe grip dynamometer (T.K.K. 3362; Takei Scientific Instruments Co., Ltd., Niigata, Japan). This assessment tool has been shown to be valid and reliable^39,40^. Measurements were taken with the knee bent at a 90º angle while the participant was seated in a chair in a neutral ankle position, neither dorsi- nor plantarflexed, nor internally or externally rotated^41^. Both feet were measured twice, and the maximum value (kg) was used.

A sit-up performance test was conducted using the method described by Abe et al.^42^. This assessment tool has been used to assess core muscle strength and endurance^26^. The participants were positioned on a mat in a supine position with the knees bent at an angle of approximately 90º. The participants performed a complete sit-up, reaching an upright position with the elbows touching the thighs and then returned to the supine position. The number of repetitions completed in 30 s was recorded.

#### 30-s stand test (CS-30)

The CS-30 was performed using the method described by Jones et al.^43^. The participants were seated in a chair (height, 40 cm) with their arms crossed over the chest. Rising to a standing position with the knees completely extended, then returning to a seated position was considered as one cycle. Participants were instructed to complete as many cycles as possible within 30 s after the starting signal had been given. The number of sit-stand-sit cycles completed in 30 s was counted.

#### Sit-and-reach test

The sit-and-reach test was performed using a digital sit-and-reach test box (T.K.K. 5412, Takei Scientific Instruments Co., Ltd., Niigata, Japan). This assessment tool has been shown to be valid^44^. The distance covered by the fingertips while reaching was measured. Measurements were conducted twice, and the maximum observed distance (cm) was used.

#### One-leg stance test

The one-leg stance test time was assessed using a digital stopwatch. The length of time the participant could maintain a one-leg stance with their eyes open was measured^39^. Two measurements were taken for each leg, and the maximum length of time was used for further analyses. The maximum time for the one-leg stance test was restricted to 120 s.

#### Fastest gait speed

The fastest gait speed was assessed using a digital stopwatch on an 11-m walkway divided into two 3-m practice sections flanking a 5-m measurement section. At the start of the measurement, the participants were verbally instructed to walk ‘as quickly as possible’, and their walking time was measured. The gait speed (m/s) was calculated using the recorded time (s).

#### Timed up-and-go test

The timed up-and-go (TUG) test was performed while walking ‘as quickly as possible’, as explained by Shumway-Cook et al.^45^. At the start signal, the participants rose from a seated position in an armless chair (height, 40 cm), walked around a pole 3 m in front of them, and returned to sit in their original chair. This process was timed using a digital stopwatch. Measurements were taken twice, and the shortest time was used.

### Cognitive function

Cognitive function was assessed using the MMSE. The MMSE is a widely used general test for assessing cognitive function. The examination comprises 11 items with a maximum possible score of 30 points^46^. A score of ≤ 23 points indicates cognitive impairment^47,48^.

### Statistical analysis

Participants with FEV_1_/FVC ≥ 70% and a %FEV_1_ < 80% were assigned to the PRISm group, whereas those with FEV_1_/FVC ≥ 70% and %FEV1 ≥ 80% were classified into the normal respiratory function group. Propensity score matching was used to identify control subjects with normal spirometry (n = 80) while minimizing the effects of confounders during comparisons with the PRISm population (n = 80).

Covariates for propensity score matching were determined to be age, sex, smoking history, and SMI. Participant attributes and physical functions were then compared between the groups. Student’s t-tests were performed for continuous variables, and chi-square tests were performed for nominal variables. The φ and d values were calculated as measures of the effect size. Logistic regression analysis was performed using the PRISm group as the dependent variable. Previous studies have found that PRISm was associated with age, sex, BMI, diabetes mellitus, smoking history, and %FVC^5,7,15,16^. Therefore, we created a logistic regression model that incorporated indicators with an φ value of ≥ 0.1 and a ‘d’ value of ≥ 0.2 as independent variables, after excluding the covariates in the propensity score matching. Lastly, a ROC curve was drawn using the results of the logistic regression analysis and indicator cut-off values determined using the Youden Index. All tests were two-tailed and used a statistical significance level of 5%. Statistical analyses were conducted using SPSS version 24 software.
